# Patient experiences and preferences for antiretroviral therapy service provision: implications for differentiated service delivery in Northwest Ethiopia

**DOI:** 10.1186/s12981-022-00452-5

**Published:** 2022-06-27

**Authors:** Yihalem Abebe Belay, Mezgebu Yitayal, Asmamaw Atnafu, Fitalew Agimass Taye

**Affiliations:** 1 Department of Public Health, College of Health Sciences, Debre Markos University, Debre Markos, Ethiopia; 2 Department of Health Systems and Policy, Institute of Public Health, College of Medicine and Health Sciences, University of Gondar, Gondar, Ethiopia; 3 Department of Accounting, Finance, and Economics, Griffith University, Brisbane, Australia

**Keywords:** Experience, Preference, Patients, Antiretroviral therapy, Northwest Ethiopia

## Abstract

**Background:**

Understanding the experiences, needs, preferences, and behaviors of people living with HIV (PLHIV) are critical to tailor HIV treatment. However, there is limited empirical evidence in Ethiopia on the views of PLHIV regarding their experiences with current antiretroviral therapy (ART) services and preferred models of HIV treatment. Hence, this study aimed to explore the patients’ experiences of taking medications and preferences for ART service provision in Northwest Ethiopia.

**Methods:**

A phenomenological study design was employed. In this study, stable and 18 years old and above PLHIVs, who had been using ART service at four public hospitals and two health centers in East Gojjam, West Gojjam and Awi zones, and Bahir Dar city administration of Amhara National Regional State, Northwest Ethiopia, were purposively selected. Fifteen in-depth interviews were conducted from July 2021 to September 2021 to collect data. ATLAS.ti version 9 software was used for coding translated transcripts. A thematic analysis approach was employed.

**Findings:**

Participants in this study had reported positive and negative experiences in receiving ART services and also varied preferences toward ART service features. The study identified five themes on experiences for ART service and 15 attributes of ART service characteristics. The identified themes were stigma, time, availability of drugs and providers, costs for clinic visits, and provider-patient interaction. The fifteen attributes were buddy system, ART refill (individualized or group), ART packaging and labeling, drug formulation and administration, ART room labeling, distance, location of service, preferences on involvement in treatment decision-making, the person providing ART refills, provider’s attitude, spatial arrangement of ART room, time of health facility operation, time spent at clinics, and total cost of the visit.

**Conclusions:**

The results raise awareness for the positive and negative experiences of patients informing us about barriers and supporting factors in ART service provision. They open up the potential for HIV treatment service improvement. The preferences of PLHIVs toward ART service delivery features were heterogeneous. Policy and program efforts should tailor ART services that suit patients’ needs and priorities in Ethiopia. Future research should further assess the reasons for patients’ distrust of the community ART delivery models.

**Supplementary Information:**

The online version contains supplementary material available at 10.1186/s12981-022-00452-5.

## Background

Global progress is being made towards achieving the Joint United Nations Programme on HIV/AIDS (UNAIDS) 95-95-95 HIV targets: 95% of people with HIV diagnosed, 95% of those diagnosed on treatment, and 95% of those on treatment virally suppressed [1]. Since 2015, the World Health Organization (WHO) has endorsed a differentiated service delivery(DSD) approach to provide HIV services to support reaching the UNAIDS targets by 2030 [2]. The DSD is operationalized by adjusting the frequency of visits, the location of service delivery, the type of healthcare workers, and the package of services according to the needs of different groups of people living with HIV (PLHIV) [3].

Group models administered by healthcare staff; group models managed by clients; individual models based at facilities; and out-of facility individual models are the four types of differentiated service delivery models for HIV treatment [4]. Groups managed by healthcare workers are people living with HIV, defined as those established on ART who meet at a defined time, either at the facility or in the community, and are facilitated by a healthcare worker (including lay workers). Groups managed by clients are groups of people living geographically close who meet at an agreed community location and nominate a member to collect ART for the group from the facility on a rotating basis. This member then distributes ART to the group at the agreed community location. Common examples include community adherence groups, community ART refill groups, and community client-led ART delivery [4].

Individual models based at facilities are commonly known as fast-track or quick pick-up and go beyond extending the ART refill duration. Assigning a specific place (such as direct pick-up from a pharmacy) and time for ART refills that do not involve consultation with a healthcare worker for clinical review or scripting minimizes time spent at the clinic. Individual models outside facilities vary according to where in the community services are provided and by whom. They can be divided into fixed community points (including private or community pharmacies), mobile outreach ART delivery, and home delivery [4].

The DSD models for antiretroviral treatment (ART) for HIV are being scaled up throughout sub-Saharan Africa(SSA) in the expectation that they will better meet the needs of patients, improve the quality and efficiency of treatment delivery and reduce costs while maintaining at least equivalent clinical outcomes [5, 6]. In response to COVID-19, adaptations to DSD for HIV treatment have resulted in quick policy change and, in certain circumstances, acceleration of implementation in SSA. This was done to ensure continuous access to ART and to reduce the number of visits to health facilities [7]. Ethiopia has also adopted the four DSD models (appointment spacing, fast track ART refill, health extension professional, and peer-led models) since 2017 for stable PLHIV taking ART [8].

A successful DSD model for HIV treatment program demands understanding each client’s experiences, needs, preferences, and behaviors, and then designing interventions that reach them where they are and deliver services in ways that meet their needs [9, 10]. A meta-synthesis of global studies by Hall et al. identified stigma and discrimination, fear of HIV status disclosure, task shifting to lay health workers, human resource and institutional challenges, mobile health, family and friend support, intensive case management, and relationships with caregivers as facilitators or barriers for retention in HIV care [11]. A systematic review of global studies by Belay et al. found that patients preferred facility-based ART service over community service, shorter travel distance, reduced waiting time, less frequent clinic visits, good providers’ attitude, having shared decision making with providers, and individualized models [12].

Local research reporting patient perspectives on ART service and their preferences is critical in further program design of DSD models in Ethiopia. However, there is limited research evidence in Ethiopia on the views of PLHIV regarding their positive and negative experiences with current ART service and their preferred models of HIV treatment. Therefore, this study aimed to explore patients’ experiences of taking medications and preferences for ART service provision characteristics in Northwest Ethiopia to aid policymakers, program managers, and practitioners as they implement and scale up DSD models.

## Methods

### Study approach

This qualitative study with the phenomenological design was conducted as part of the larger discrete choice experiment study. We adopted the phenomenological approach because it focused on the lived experiences of HIV-positive patients when seeking HIV treatment services from the perspective of the patient [13]. Data were collected between July 2021 and September 2021.

### Study setting

The study was conducted at the HIV clinics of four hospitals: Felege Hiwot Comprehensive Specialized Hospital (FHCSH), Debre Markos Comprehensive Specialized Hospital (DMCSH), Finoteselam General Hospital (FGH), and Dangila Primary Hospital (DPH); two health centers: Debre Markos Health Center (DHC) and Bichena Health Center (BHC) in East Gojjam, West Gojjam and Awi zones, and Bahir Dar city administration of the Amhara National Regional State, Northwest Ethiopia.

### Sampling and recruitment

A purposive selection of ≥ 18 years old stable ART clients in the public health facilities of East Gojjam, West Gojjam and Awi zones, and Bahir Dar city administrative of the Amhara region was done. Stable ART clients were defined as per the Ethiopian HIV care and treatment guideline as those ≥ 18 years of age, who have received ART for at least one year and have no adverse drug reactions that require regular monitoring, are still on first line regimen, have no current illnesses or pregnancy and are not currently breastfeeding, have good understanding of lifelong adherence, and have evidence of treatment success (current viral load measurement with in the last 12 months below 1000 copies/mL or rising CD4 cell counts or CD4 counts > 200 cells/mm3 in the absence of viral load determination [14]. We recruited participants from different facilities based on a load of patients taking ART and facility type (tertiary, general and primary hospitals, and health centers).

In-depth interviews with adults on ART who were virally suppressed and eligible for DSD on the date of data collection [2] were conducted. The facility staff members who were already aware of the study were requested to support the recruitment of patients among those that were visiting for a regularly-scheduled ART appointment declared to be eligible for DSD models on the date of interview. The sample size was determined based on data saturation. In conducting interviews, scholars have found that data saturation often occurs within the first twelve interviews while meta-themes might appear after six interviews [15]. Fifteen (FHCSH = 5, DMCSH = 3, FGH = 2, BPH = 1, DMHC = 2, and BHC = 2) participants were approached for interviews. There was no new information that was obtained at the 13th interview and hence a saturation point was believed to have been achieved at this point. This number also fulfills the required sample size for phenomenological approaches [16].

### Data collection

A semi-structured interview guide was developed and used for data collection. The guide consists of questions about the patients’ experiences with current ART service and preferences towards features of ART service provision (Additional file 1). Interviews were conducted by the principal investigator (YAB) in the Amharic language. The interview guide was pre-tested among purposively selected participants at DMCSH before actual data collection. Data were collected from participants in a private room in the health facilities. The interviews were audio-recorded and lasted between 30 and 105 min. Transcription of audio files and initial analyses were carried out in parallel with the data collection allowing the study principal investigator to get an insight into theoretical saturation. The interviewer wrote field notes during and after the interviews.

### Data analysis

Participants’ responses were transcribed verbatim. Then, the transcripts translated into English were imported into ATLAS. ti version 9 software for coding and were analyzed using a thematic analysis approach. Our analysis followed a hybrid approach of inductive and deductive coding. Initial deductive coding was based on the fixed topics from the literature review [12], study objectives, and interview guide followed by an inductive, data-driven generation of new codes. Familiarization with the data was done by reading and rereading the transcripts followed by developing the codebook. Memos were written during the coding process to capture impressions and to facilitate the identification of themes. The study was reported according to the consolidated criteria for reporting qualitative studies [17] (Additional file 2).

### Trustworthiness of the study

Trustworthiness in qualitative research is achieved by enhancing credibility, dependability, confirmability, and transferability [18]. The credibility of the study was enhanced by spending more time with participants in individual interviews until data saturation was reached. There was also iterative questioning (the use of probes to elicit detailed data and returning to matters previously rose by an informant and extracted related data through rephrased questions) to enhance credibility. Dependability was enhanced by maintaining an audit trail by keeping all copies of notes, transcribed and recorded data for future use, including supplying participants with researchers’ personal and academic information for contact or explanation at any time.

Confirmability was enhanced by conducting a pilot study, which served as a pre-test to the interview schedule, and interviewing skills from 3 participants at DMCSH helped to refine the study methods. The results of the pilot were not a part of the report presented in the final study. Transferability was enhanced using a non-probability purposive sampling method to collect data from participants. We also supported transferability via rich descriptions and verbatim quotations from the transcripts.

### Ethical approval

This study received ethical approval from the Institutional Review Board of the University of Gondar (approval number V/P/RCS/05/762/2021). A formal letter obtained from Amhara Public Health Institute was given to each health facility to get permission and cooperation. After briefing the purpose of the study, written informed consent was taken from each participant before the data collection.

### Findings

### Participant characteristics

A total of 15 PLHIV who were on ART were interviewed. The mean age of the participants was 38.8 ± 3.59 years. The mean duration of ART intake was 10.1 ± 1.43 years. There were 10 females, 4 were married, and 8 had attended education. Moreover, 10 were employed (Table 1).

**Table 1:** Socio-demographic characteristics of participants (n = 15), in Northwest Ethiopia, 2022

Characteristic	Percent (number)
Age (years)	
Mean (SD)	38.8 (3.59)
Range	22,72
Gender	
Female	66.67 (n = 10)
Male	33.33 (n = 5)
Education	
None	46.67 (n = 7)
Primary	13.33 (n = 2)
Secondary and higher	40.00 (n = 6)
Marital status	
Married	26.67 (n = 4)
Divorced	46.67 (n = 7)
Never married	26.67 (n = 4)
Employment	
Unemployed	33.33 (n = 5)
Unskilled employment	46.67 (n = 7)
Skilled	20.00 (n = 3)
Duration on ART (years)	
Mean (SD)	10.1 (1.43)
Range	1,20

n: number; SD: Standard Deviation

Key qualitative findings were described below and organized according to the two major themes associated with the patients’ experiences with ART service and preferences for ART service delivery characteristics expressed across all participants. An overview of patients’ experiences with ART service and their preferences for ART service provision characteristics identified from the interview were presented in Tables 2 and 3 respectively. Illustrative comments from interviewed participants were included as appropriate.

**Table 2:** Categories, themes, and supporting themes for participants’ experiences with antiretroviral therapy in Northwest Ethiopia, 2022

Categories	Themes	Supporting quotes
Perceived/anticipated stigma	Stigma	*“I take my drug in a hiding place when my child sleeps since she didn’t know about my status. I always come from a remote area which takes 1 h by bus since there is still a stigma in our community. My social life and work could be affected if I get service in my locality”. (33 years old female)* *“No one knows about my HIV status except my wife hence I didn’t experience discrimination from others”. (56 years old male)* *“My husband’s family members considered me as doing some evil activity on him. They advised him to stop the HIV drug and rather go to a traditional healer”. (57 years old female)* *“I have an experience of discrimination by a woman with a rental dorm. She refused to accept me to rent in her house as a result of knowing my HIV status”. (27 years old female)* *“I have found a discriminatory action by the gatekeepers. They [gatekeepers] openly asked me the reason for the entry to the hospital in front of many people requesting to enter the hospital during COVID-19 movement restriction”. (40 years old male)*
Enacted stigma from family		
Enacted stigma from the community		
Enacted stigma from the healthcare setting		
Waiting time	Time	*‘‘The main challenge was the long queues here. You may wait for a long time or even postpone to the next day by taking the emergency drugs only’’. (22 years old male)* *‘‘I didn’t see the clinic operating on the weekends and before 2:30 in the morning or after 11:30 in the afternoon from Monday to Friday’’. (40 years old female)* *‘‘It takes me 2 h of travel by bus’’. (40 years old female)* *“I was visiting the facility every month for 7 years”. (22 years old male)* *“The laboratory test clashes with my education schedule since the facility always tell me to come in the morning where there may be a class in the school”. (22 years old female)* *‘‘I am still frustrated with disclosing my status to others. I tried to request one of the providers here and she send me my drugs *via* postal service since I was not able to come on the appointment date due to a clash with my work’’. (33 years old female)*
Facility operation time		
Travel time		
Frequency of health facility visit		
Time convenience with school and work		
Costs of transportation	Costs per clinic visit	*‘‘I come from a remote area which takes 1 h by bus with 60 birr cost. Since there is still a stigma in our community, I am forced to come here for getting ART service. I may take paying fewer costs for a taxi if I attended in my locality’’. (33 years old female)* *‘‘I pay for the transport and food costs when I come here. But, I do not pay for drugs at this hospital since I have no opportunistic infections’’. (40 years old male)* *‘‘I have discussed with my providers to give me drugs with additional stock at least for 10 days in addition to the usual prescription to avoid work inconvenience’’. (33 years old female)*
Costs of drugs for opportunistic infections		
Additional costs including food		
Cost of missing work when seeking care		
Attitudes and behaviors of healthcare workers	Provider–patient interaction	*“The providers are kind enough to treat us. Some providers have HIV and help us properly. They [providers with HIV] counsel us very helpful in a good manner. It is like knowing about the hungry status of someone by remembering their own hungry experience. I always want to contact them”. (33 years old female)* *‘‘I found most providers are good at providing services including counseling. But some providers are not showing bright faces, do not give adequate counseling and simply order to take drugs for 3 or 6 months, and even do not close the door which affects our privacy’’. (38 years old male)*
Counseling		
Drug availability	Drug and provider availability	*‘‘I am satisfied with the drug supply in this hospital’’. (40 years old female)* *‘‘The current service is not bad. It could be better if there are an adequate number of providers here’’. (22 years old male)*
Providers’ availability

**Table 3:** Summary of attribute labels, lay terminologies, labels of plausible levels, and illustrative quotes in Northwest Ethiopia, 2022

Attribute label	Lay terminology	Illustrative quotes	Labels of plausible levels
Participants/others seen at the same visit	Individual appointment versus an appointment that includes other patients	*‘‘I prefer the individual-based service to avoid disclosing my status when I form groups and take drugs together with the other clients’’. (40 years old male)* *‘‘I prefer the individualized service to avoid clashing with other group members regarding the scheduled time to meet’’.* (72 years old male)	Individual
		*‘‘I prefer the group-based service since we have an opportunity of sharing ideas’’.* (22 years old male) *‘‘I prefer the group-based service since it helps us to support one another by strengthening our social interactions. It also saves our time to come here individually and use our time for our work by taking drugs in the village’’.* (40 years old female)	Group
ART packaging	Patients stated preferences for ART packaging attributes	‘‘I also expect that the drug package should be changed since the current bottle-based package created discrimination by others as they could easily identify it. The bottle should be changed so that the container can handle many drugs and even we can put it in our pocket to avoid the direct advertising act of the current package. (33 years old female)	Need for change of drug package
		*‘‘I don’t have any problem with the drug packaging including the bottle sound. I use festal to carry my drugs since there is a large number of drugs. But, there may be stigma from the community to carrying a large volume of drugs for some clients’’.* (57 years old female)	No need for a change of drug package
ART room labeling	Branding of ART clinics	‘‘I prefer the clear labeling of the ART room to help me in identifying the service delivery room’’. (72 years old male)	Clear labeling of ART room
		*‘‘I prefer the non-posting of the room to avoid discrimination by others while I get into this service room’’.* (22 years old male)	No clear labeling of ART room
Buddy system	Means someone can pick up clients’ meds if they are not due for a provider visit	*‘‘I prefer having the other person to assist me in drug-taking since there may be some time inconvenience for me to come here’’.* (33 years old female) *‘‘It would be better to have other persons who assist in case of bedridden or paralyzed cases’’*. (72 years old male)	Buddy system in place
		‘‘I prefer to take drugs myself since I will not be happy if another person brings drugs for me that may not be trusted like me’’. (40 years old male) ‘‘I prefer to come physically here to be checked about my health status and whether the drug is working or not. It should not be thought of simply taking drugs from here’’. (25 years old female)	No buddy system in place
Distance from residence to a clinic	The proximity of a health facility to home	*‘‘I prefer the clients get the service in a near place. It reduces time, costs for transportation and could help to engage in other work activities’’.* (38 years old male)	Near
		*‘‘It is my interest to come from a remote place since there is still a stigma in our community while getting service in my locality’’.* (33 years old female)	Far
Drug administration	Form of drug administration	*‘‘It could be better if there is a vaccination like for the other diseases or an injection that could be used at least for one year’’.* (25 years old female)	Pill based
			Injection
Drug formulation	Novel drug formulation methods	*‘‘I would be happy if there is a curable drug for us or drugs with fewer side effects or some additives in the drug that boosts the client’s immunity like vitamins’’.* (33 years old female) *‘‘I expect there is a drug that builds our body like proteins in the drug so that you will be fat and similar to other HIV naïve people’’.* (40 years old male)	Drug with additives and/or protein
		*‘‘The current pill-based drug has side effects on the stomach. It needs reconsideration’’.* (38 years old male)	Drugs with fewer side effects
		*‘‘If possible I prefer if there is a permanent cure for HIV. I would stop visiting this hospital if there is a cure for HIV’’.* (38 years old male)	Effective or curable drug
Frequency of receiving ART refills	Frequency of routine visits for ART refill	*‘‘I prefer to come every three months here. It could help me to be checked my health status regularly. If I take the drugs every 6 months or yearly, I may be sick with an opportunistic infection and my viral load could be increased due to the long time to check my status by the providers’’. (57 years old female)*	Every 3 months
		*‘‘I prefer to come every 6 months per year. But we can come here if we become sick in between the appointment dates’’.* (38 years old female)	Every 6 months
		*‘‘I prefer to come once per year if I am healthy. It avoids transport costs and losing our daily work there at our locality. I may come at any time here if I have illness in between’’.* (32 years old female)	Yearly
Labeling of ART package	Labeling for medicines	*‘‘I don’t care about it [labeling of drug package]. There may be some others who may discriminate against us [clients with HIV on ART] when they [others] see the package and read the labeling of ARV drugs on the boxes’’. (38 years old female)*	Medicine labels must be clear
		*‘‘I think there should not be the labeling of the drug package to avoid being readable by other people than me like my child. She may search *via* Google and know about her status’’.* (33 years old female)	Medicine labels must not be clear
Location of ART service delivery	Preferred place of service for patients	*‘‘I prefer the facility-based service since there may be a problem that will occur to give service at the community level by the current level of understanding. Providers may break confidentiality to let know others know about my status.’’.* (33 years old female) *‘‘I prefer the facility-based ART service since the clients could get their providers in time and get appropriate service here. I have a concern there in the community that the providers may not deliver service like the providers in the facility. I may not be at home on the appointment date or I may be not aware of the exact appointment date there and I may create a problem for my providers in this case’’.* (72 years old male)	Health facility
		*‘‘I prefer the community-based service since it [community-based service] saves time and lets clients know each other’’.* (57 years old female) *‘‘I would be happy if the service is given at the community level since it avoids a long queue at this hospital and waiting time’’.* (40 years old female)	Community
Preferences on involvement in treatment decision-making	Preferred participation and roles of patient and provider in treatment decision-making	*‘‘I prefer a joint decision to select the model. There could be sharing of each idea by the clients and the providers. There should be an agreement between the two entities. There may be damage if one of the two simply selects the model’’.* (25 years old female)	Make treatment decisions together with their provider
		*‘‘I couldn’t decide my model of choice. The provider should select the appropriate options since he knows the benefits and harms of this approach. The clients shouldn’t select the options for them rather the providers should select them’’.* (40 years old male)	Providers making treatment decisions entirely by themselves
		*‘‘I prefer to select the model of my choice since I have a reason to choose from depending on my context. The provider should not decide for me’’.* (72 years old male)	Patients make treatment decisions on their own
The person providing ART refills	The person who delivers ART refill services	*‘‘I prefer the healthcare workers since they have their training. They can give the drugs by knowing the benefits and the harms. But in the case of the HEW or peer leaders, they lacked the appropriate knowledge and even they may wrongly exchange our drugs. I never trust them in this regard’’.* (27 years old female) *‘‘I prefer the healthcare workers since they are trained to identify and manage the problems that I may have by critically evaluating my health status. However, the peer leaders are similar to me in terms of knowledge and couldn’t provide drugs for me properly’’.* (38 years old male) *‘‘I prefer the healthcare workers to deliver the ART service. I don’t accept the peer leaders distributing our drugs since there is discrimination by the local community perceiving us having a meeting of HIV-positive people there’’.* (40 years old female)	Healthcare workers
		*‘‘I prefer the health extension workers to give me drugs since I can go to her without notifying my status’’.* (40 years old male)	Health extension workers
		*‘‘I prefer the peer leaders to bring us our drugs since they know everything and they have experience of drug-taking. They give more empathy to us compared to health extension and health care workers. Others couldn’t appreciate the context despite they have trained on the disease and the drug and give the service by reading on it’’.* (40 years old female)	Peers
Provider’s attitude	Staff attitude while in care and treatment	*‘‘My choice depends on the provider you get every visit. You may not get the provider that initially hosts you when you come here at your appointment. There is a difference in the kindness of the providers while giving service to us. I had one female provider who treats me with a bright face. Only a few providers are showing their good approach to us. There may be a behavioral problem by providers due to an increased load of clients’’*. (38 years old male)	Rude
			Nice
The spatial organization of service	Choice of separate or integrated service rooms	*‘‘I prefer the separate building of the ART room to avoid the associated stigma if the ART room is available with other service rooms of the health facility’’.* (72 years old male)	Separate service room
		*‘‘I prefer the service should be given with another service in the same room to avoid HIV status. The providers should treat them accordingly based on the clients’ situation instead of a separate service for ART’’.* (33 years old female) *‘‘I would be okay if the service is connected with other services. It is similar to other services in the hospital. I have raised a question for myself why the service room is isolated from other service delivery rooms in the same facility’’.* (22 years old female)	Integrated HIV and other services room
Time of facility operation	Health facility’s opening days and hours for ART refill	*‘‘I prefer the usual working days and hours since it is enough to get the service at that time’’.* (38 years old female)	Workweek and usual hours
		*‘‘I prefer the weekend-based service since I come here freely to take drugs these days’’.* (22 years old female)	Weekend service only
		*‘‘I prefer the extra facility opening time especially Saturday and Sunday in addition to the usual working hours and days. I am not comfortable with the facility openings before 2:30 in the morning and after 11:30 in the afternoon since we do not come to this hospital at such times’’.* (40 years old female)	Workweek and weekend days
		*‘‘I choose a 24-h ART service in this hospital since we may be sick at any time. We get the service in case of difficulty if the facility is open all days including night’’. (25 years old female)*	24 h of the day at any time of the week
Time spent at clinics in ART pick-ups	Waiting time for registration, consultation, and ART pick-ups	*‘‘I would prefer to get service in the shortest time since I am busy either going to work or school. But, if the service demands to wait for more, I would wait here instead’’.* (22 years old male)	Less waiting time
		*‘‘I prefer to wait a long time to learn from my providers about the drug and the related things. I also want to talk with other clients in this hospital. I would be happy when I come here and see other clients taking drugs like me. I become frustrated when I am alone in the rural area of my home’’.* (40 years old female)	More waiting time
The total cost of a visit	The total cost of the visit includes transportation, direct medical costs (e.g., consultation or booking fee, lab costs if not available at a public facility, non-ARV drug costs), and costs of food	‘‘I will pay whatever transport cost I have been requested to come here. I have health insurance and do not pay other payments in this hospital’’. (40 years old female)	Willing to pay the cost of transport
		‘‘I believe that the free service that is currently being done is a good one. I recommend a balanced payment or a free service for those clients unable to pay for it [service]’’. (33 years old female) ‘‘I want there should be a free service related to HIV and other illnesses since living nowadays is becoming hard and there are some clients who live in poverty unable to cover the costs of medications’’.(27 years old female) ‘‘I prefer the service free of charge. There should be a special arrangement from the facility to cover the cost of transportation for those clients who do not generate income like old persons’’. (25 years old female)	Free and/or subsidized cost

### Participant experiences with antiretroviral therapy service

Participants in this study had reported both positive and negative experiences when they received care or treatment from the ART clinics. Stigma, time (waiting, facility opening, travel, frequency of health facility visit, and time convenience with school and work), drug and provider availability, costs per clinic visit, and provider-patient interaction were the themes that emerged regarding the participants’ experiences with ART and were highlighted in the following section as follows:

#### Stigma

Perceived/anticipated stigma following HIV status disclosure was quite prevalent among all PLHIV in the study. Maintaining secrecy or limiting disclosure of HIV status appeared to be a protective strategy among many participants. *“I take my drug in a hiding place when my child sleeps since she doesn’t know about my status. I always come from a remote area which takes 1 h by bus since there is still a stigma in our community. My social life and work could be affected if I get service in my locality”* (33 years old female). Some participants reported experiences of stigma from family, community, and healthcare settings. However, no participant mentioned an experience of stigma by their healthcare providers.

#### Time

The participants in this study mentioned their experiences with waiting time, facility opening, travel time, frequency of health facility visits, and time convenience with school and work. Regarding waiting time, many participants reported that there was a large crowd of clients at the ART clinics in the past. The large crowds in their opinion resulted in long waiting hours before consultations or drug refills. *“It takes 1–2 h to wait here depending on the number of clients being served. We are getting our cards from the main card room with other HIV-negative individuals creating a big concern for us” (38 years old female)*. Some participants however reported waiting less time at a health facility. Under facility operation time, the study participants frequently mentioned late clinic opening and early closing hours were issues frustrating patients in accessing care. *‘‘It could be okay to return to work if we get the service before 2:30 in the morning. But the providers are losing our time here by starting the service at 3:00 instead of the legal 2:30’’ (40 years old male).* One participant, however, said that the providers used to start working early in the hospital.

Regarding travel time, clients reported varied experiences in the duration of travel time from home to a facility. Many participants highlighted less travel time whereas some participants reported more travel time from home to a facility. Clients also mentioned their experience of health facility visit frequency as more frequent of health facility visit schedules in the past. *“I was visiting the facility every month for 7 years”* (22 years old male). Other participants however reported less frequent visits. Participants also reported regarding their experiences on time convenience of the services, and some participants reported time inconvenience with school and work. *“The laboratory test clashes with my education schedule since the provider always tell me to come in the morning where there may be a class in the school”*(22 years old female).

#### Costs per clinic visit

The participants in this study mentioned that only ARV medication and a few laboratory investigations were free. The participants highlighted that they used to cover the costs of transportation, drugs for opportunistic infections, and additional costs including food. Some participants expressed their concern about the expensiveness of the cost of transportation and accommodation when coming from remote areas due to fear of stigma and discrimination in the community. Some participants also reported that they used to miss work when seeking care.

#### Drug and provider availability

No respondents in this study reported experiencing a complete ARV drug stock-out at the health facilities. Most participants reported a shortage of health care providers in the ART clinics. *‘‘There should be additional providers employed in the ART room since the number of clients is many hence to avoid waiting for a long time here’’ (22 years old female).*

#### Provider–patient interaction

Most participants mentioned the good attitudes and behaviors of healthcare workers (HCWs) towards them, which they said have encouraged them to continue accessing HIV care and treatment. Some patients reported encountering HCWs who were disrespectful, non-caring, and providing inadequate counseling.

### Participant preferences for antiretroviral therapy service characteristics

Fifteen attributes (themes) with respective attribute levels were identified in the thematic analysis. Attributes and attribute levels were identified from the transcripts and prominent participants’ quotes were directly extracted to illustrate each attribute and attribute level (Table 3). The identified attributes were highlighted in the following section.

#### Buddy system

The majority of participants preferred having physical contact with the health facility provider over having someone assisting in collecting their pills. The reasons cited were the need for health status check-up, weight check-up, counseling, checking the effectiveness of taking the drug, and lack of trust in someone collecting drugs. ‘‘*I prefer to come physically here to be checked about my health status and whether the drug is working or not. It should not be thought of simply taking drugs from here*’’ (*25 years old female*). There were however some participants who preferred someone to take drugs for them from the facility on their behalf in case of time inconvenience on the date of appointment, bedridden condition, and engage in their work activities on the facility visit days.

#### Individualized or group ART refill service

The majority of the participants preferred individualized ART refill service to group-based service due to a need for maintaining privacy and confidentiality, avoiding clashing with other group members, weight check-ups, and fear of drug change, and getting appropriate service. Some participants on the other hand chose the group form of ART service for reasons of experience sharing, strengthening social relationships, and using the time for work. *‘‘I prefer the group-based service since it helps us support one another by strengthening our social interactions. It also saves our time to come here individually and use our time for our works by taking drugs in the village’’* (*40 years old female*).

#### ART packaging and labeling of drug package

Nearly all participants indicated that they would prefer a change of drug package because the box and bottle are large and easily identifiable by others. Regarding the labeling of drug packages, the majority of participants preferred the medicine labels must not be clear to avoid disclosure of HIV status. Some preferred however the medicine labels must be clear for easy identification by clients.

#### Drug formulation and administration

There was heterogeneity in participants’ preferences for the aspects of drug formulation and administration. Some participants preferred the ARV drug formulation with additives and/ or protein. Some participants preferred the effective or curable drug formulation. Some other participants also preferred an injectable form of ARV drugs.

#### ART room labelling

The majority of participants preferred the clear labeling of the ART room to help them in easy identifying of service delivery room. Some of the participants however preferred the non- labeling of the ART room to avoid notifying their HIV status. *‘‘I prefer the non-posting of the room to avoid discrimination by others while I get into this service room’’.* (22 years old male).

#### Distance from residence to a clinic

Most of the participants preferred a near distance from their homes to the health facilities. *‘‘I prefer to get the service in a near place. It reduces time, costs for transportation and could help to engage in other work activities’’* (*38 years old male*). Some however preferred a far distance from home to the health facility due to the concerns of privacy and confidentiality.

#### Frequency of receiving ART refills

The study participants had a variety of preferences for the frequency of receiving ART refills. The majority of them preferred having a facility visit every 6 months for ART refills. *‘‘I prefer to come every 6 months per year. But we can come here if we become sick in between the appointment dates’’* (*38 years old female*). Some participants chose to attend the health facilities every 3 months. Some of the participants preferred yearly visit for ART refills.

#### Location of ART service

The majority of the participants preferred a facility-based ART service over a community-based service including home delivery. The common reasons mentioned were concern about confidentiality, getting health investigation, getting timely and appropriate service. *‘‘I prefer the facility-based ART service since we could get our providers in time and get appropriate service here. I have a concern there in the community that the providers may not deliver service like the providers in the health facility’’* (72 years old male). Some participants chose the community-based ART service due to reasons of time-saving, clients knowing each other, and avoiding long queues at a health facility. *‘‘I prefer the community-based service since it [community-based service] saves time and lets clients know each other’’* (57 years old female).

#### Preferences on involvement in decision-making of differentiated HIV treatment model option

The majority of participants preferred to make treatment decisions on their own. Some participants preferred the provider’s entire treatment decision on their behalf. Some other participants attached more importance to reaching a consensus and having a shared responsibility in decision-making. *‘‘I prefer a joint decision to select the model. There could be sharing of each idea by the clients and the providers. There should be an agreement between the two entities. There may be damage if one of the two simply selects the model’’* (*25 years old female*).

#### The person providing ART refills

Most of the study participants preferred receiving the ART refills from the healthcare workers at the health facility due to their knowledge, training, and concern for confidentiality. *‘‘I prefer the healthcare workers since they have training. They [healthcare workers] can give the drugs by knowing the benefits and the harms. But in the case of the health extension workers [HEWs] or peer leaders, they [HEWs or peer leaders] lacked the appropriate knowledge and even they may wrongly exchange our drugs. I never trust them in this regard’’* (*27 years old female*). One participant preferred the HEWs for concern of maintaining privacy. One participant also chose to get the ART refill by the peers for sake of being understood well. In Ethiopia, two Health Extension Workers (HEWs) are assigned per Kebele, which is the lowest administrative unit of the government structure with an average of 1000 households and approximately 5,000 people. The HEWs provide services at the health post and in the community [19].

#### Provider’s attitude

All participants in this study put a strong preference for having nice over rude providers. They valued more on the empathy and positive attitude of their providers.

#### The spatial arrangement of the ART room

Most participants preferred a separated ART clinic from main health facility buildings for sake of privacy. *‘‘I prefer the separate building of the ART room to avoid the associated stigma if the ART room is available with other service rooms of the health facility’’* (*72 years old male*). Some participants however prefer the shared space of services in the main health facility buildings for reasons of hiding HIV status and considering HIV service similar to other services.

#### Time of the health facility operation

The majority of participants in this study preferred getting ART service during the workweek (Monday to Friday) in the usual hours (2:30–6:30 morning and 7:30–11:30 afternoon). *‘‘I prefer the usual working days and hours since it is enough to get the service at that time’’* (*38 years old female*). Some preferred to get the service during the work week with weekend days. Others chose to obtain the service 24 h of a day at any time of the week. One participant preferred weekend service only.

#### Time spent at clinics in ART pick-ups

Most study participants preferred less waiting time at the ART room in ART pick-ups including registration, consultation, and pharmacy dispensing, cited saving time for work or school as the main factor. *‘‘I would prefer to get service in the shortest time since I am busy either going to work or school. But, if the service demands to wait for more, I would wait here instead’’* (22 years old male). Few participants however chose to wait more time in the facility due to a need for adequate time to discuss with providers and talk with peers in the facility.

#### The total cost of the visit

Nearly all participants preferred either a free or subsidized cost of visit including transportation and medications other than ARV drugs. *‘‘I believe that the free service that is currently being done is a good one. I recommend a balanced payment or a free service for those clients unable to pay for it [service]’’* (*33 years old female*). One participant however was willing to pay for an additional cost of transportation to get medication.

## Discussion

In this study, participants reported positive or negative patient treatment experiences and heterogeneous preferences on the key features of ART service provision.

### Participant experiences with antiretroviral therapy service

Participant interviews revealed that HIV stigma persists. The main type of stigma evident among our respondents was anticipated stigma where they fear facing negative outcomes from others following HIV status disclosure. This finding is consistent with other recent research linking HIV stigma to low disclosure [20–23]. This type of stigma has implications for service use and ART adherence.

Participants reported long waiting times in health facilities before consultations or drug refills in the past due to overcrowding there. This is in line with a study conducted in Zimbabwe [23]. This implies the need of adopting and implementing DSD to avoid congestion of health facilities and manage patients efficiently. Participants also mentioned their experience of more frequent health facility visit schedules in the past. This has an important implication for implementing the DSD approach to reduce the frequency of health facility visits for clients.

Participants stated financial concerns about costs of transportation, drugs for opportunistic infections, additional costs including food and accommodation as well as missed work when seeking care. This is comparable with a study conducted in Iran [24] and Zimbabwe [23]. Another study in Kenya [22] also showed that older adults described the challenges they face with costs of transportation when accessing the facilities to seek HIV care services from a distant clinic. This highlights that further decentralization of ART services to lower-level health facilities and availing a free or subsidized cost for treatment of opportunistic infections are needed which in turn contributes toward the achievement of the goal of universal health coverage by 2030. Participants reported a shortage of health care providers in the ART clinics about the number of clients getting service there causing long waiting times. This has an important implication for more investment in human resources for health thereby improving the quality of ART service provided to patients in the health facilities.

### Participant preferences for antiretroviral therapy service characteristics

Participants put varied values on individualized versus group-based ART refills. The majority attached great importance to the individualized service mainly related to concerns of privacy and confidentiality. This finding is in agreement with studies conducted in Zimbabwe [23] and in Kenya [25]. This has an important implication of practical challenge in implementing and expanding the DSD models in Ethiopia.

Most of the participants preferred a near distance from their homes to the health facilities. This finding is in line with previous studies [26, 27]. This highlights a need for further decentralization of ART services to where people live and work to achieve universal access to antiretroviral treatment. Regarding the frequency of health facility visits, participants chose to have a facility visit every 6 months for ART refills. This finding is consistent with a study conducted in Kenya [25]. This suggests that multi-month scripting best aligns with patients’ preferences, an insight that can help prioritize the use of different DSD models in Ethiopia. Regarding the location of service delivery, the majority of the participants preferred a health facility-based ART service over a community-based service. This is in line with an earlier study [25]. This highlights an important implication to enhance the promotion of the community-based models as an alternative to the facility-based models hence reducing the overloading of the existing facilities with clinically stable patients.

Participants’ preferences for their involvement in treatment decision-making were varied. The majority of them preferred to make all final decisions alone. Some preferred that their doctor make all or most decisions and others preferred to share decisions with their doctor. This is comparable with a study conducted in the United States [28]. This highlights the need for communication about patients' expectations, wishes, and preferences for participation in upcoming HIV treatment decisions. Regarding the person providing ART refills, most participants preferred receiving the ART refills from the healthcare workers at health facilities than health extension workers or peers. This is in line with a study conducted in Kenya [25]. This has an important implication for providers, health system administrators, health workforce planners, and policymakers to better understand patient perspectives and design care that enhances patient satisfaction.

Participants in this study put a strong preference for nice over rude providers. This is in line with a study in Zambia [29]. This highlights enhancing policy interventions on the implementation of motivated, competent and compassionate human resources for health. Participants preferred less waiting time at the ART room during ART pick-ups. This finding is consistent with a study in Ghana [30]. This highlights a policy intervention to increase the number of health personnel at ART clinics to reduce the time HIV-positive patients spend at such facilities. Similarly, participants preferred either a free or subsidized cost of the visit including transportation and medications other than ARV drugs. This is consistent with a previous study [12]. This has policy implications for increased access to ART service to where people live and work as well as insurance coverage.

## Limitations

This study was subject to limitations. First, it is not possible to generalize the findings of this qualitative study due to its relatively small sample size. Second, we do not have data about the actual experiences or preferences of patients since these findings are based on what the interviewed participants chose to report to the interviewer. Third, the responses from the participants of this study may not be reflective of all PLHIV taking ART since we only included a group of patients attending health services in the selected sites. Finally, this group of patients had never been exposed to the existing DSD models for HIV treatment and some patients might struggle to determine their preference for a service which they had never experienced.

## Conclusions

The results of this study raise awareness for the positive and negative experiences of patients informing us about barriers and supporting factors in ART service provision. They open up the potential for HIV treatment service improvement. The preferences of PLHIVs towards ART service delivery features were heterogeneous. Policy and program efforts should tailor ART services that suit patients’ needs and priorities in Ethiopia. Future research should further assess the reasons for patients’ distrust of the community ART delivery models (led by the health extension workers and peer leaders).

## Supplementary Information

**Additional file 1:** Patient interview guide.

**Additional file 2:** Consolidated criteria for reporting qualitative studies (COREQ): a 32-item checklist.

## Data Availability

The datasets used during the current study are available from the corresponding author on a reasonable request.

## Abbreviations

AIDS: Acquired Immunodeficiency Syndrome
ART: Antiretroviral Therapy
ARV: Antiretroviral
DSD: Differentiated Service Delivery
HCW: Health Care Worker
HEW: Health Extension Worker
HIV: Human Immunodeficiency Virus
PLHIV: People living with HIV
SD: Standard Deviation
UNAIDS: The Joint United Nations Programme on HIV/AIDS
WHO: World Health Organization
