# Supplementary material for: Patient experiences and preferences for antiretroviral therapy service provision: implications for differentiated service delivery in Northwest Ethiopia
Source: AIDS Res Ther. 2022 Jun 27;19:30. doi: 10.1186/s12981-022-00452-5 (PMC9237972; doi:10.1186/s12981-022-00452-5)
Supplement: Supplementary file 1 — Additional file 1: Patient interview guide. [file 12981_2022_452_MOESM1_ESM.docx]

**Additional file 1: Patient interview guide**

**Part I**: **Patients’ experiences with antiretroviral therapy service**

1. How long you have been on antiretroviral therapy?
2. How many times do you usually visit this place in a year to collect your medication?
3. How much time does it take for you to come to this health facility?
4. How much time do you usually spend here at this clinic when you come to pick your drug refills and do your reviews?
5. What kind of health care providers do you usually meet when you come here?

(Probes: manners and the attentiveness, care and the assistance offered, respect the providers express, welcomeness, sincerity, and privacy)

1. I would like to know in terms of payment when you pay how much does it cost you even the transport you use coming here.
2. Is there someone that you usually send to collect your pills?
3. Did you ever encounter the problem of failing to collect your medication on the day which you were appointed?
4. Are there any times when you forgot to take your antiretrovirals?
5. I would like to know about the challenges you are facing which hinder you from remaining on medication?
6. How easy or difficult is it for you to come to the clinic all the time?
7. Does your clinic where you collect your medicines open early in the morning, late evening, or over the weekend?

(Probe: - Will that make a difference on the way people are treated?)

1. I would like to know if you have experienced some stigma or discrimination may be from family members, people from your community, health care providers or even your workmates.
2. Can you suggest some changes in the way antiretrovirals are provided in health facilities or in the community which may encourage people living with HIV to access and remain in care?

**Part II**: **Patients’ preferences**

1. Now I want to hear your views with regards to things that make it easier for you to continue in care.

(Probes: Location of service (facility/community), individualized or in group service, HIV care provider type, time the health facility opens, HIV care frequency, the time you spend at the health facility, amount of money you use to get your antiretroviral drugs including transport and user fees, service package, buddy system, patient involvement, relationships with providers, and shared decision making, distance, labeling of service room, service room location in relation to other rooms, drug packaging, and labeling)
